# Chikungunya Outbreak in Country with Multiple Vectorborne Diseases, Djibouti, 2019–2020

**DOI:** 10.3201/eid2904.221850

**Published:** 2023-04

**Authors:** Emilie Javelle, Franck de Laval, Guillaume André Durand, Aissata Dia, Cécile Ficko, Aurore Bousquet, Deborah Delaune, Sébastien Briolant, Audrey Mérens, Constance Brossier, Hervé Pommier, Florian Gala, Alain Courtiol, Quentin Savreux, Sébastien Sicard, Jean-Philippe Sanchez, Francis Robin, Fabrice Simon, Xavier de Lamballerie, Gilda Grard, Isabelle Leparc-Goffart, Vincent Pommier de Santi

**Affiliations:** French Aix Marseille University, Marseille, France (E. Javelle, G.A. Durand, S. Briolant, F. Simon, X. de Lamballerie, G. Grard, I. Leparc-Goffart, V. Pommier de Santi);; French Armed Forces Biomedical Research Institute, Marseille (E. Javelle, G.A. Durand, S. Briolant, G. Grard, I. Leparc-Goffart);; University Hospital Institute-Méditerranée Infection, Marseille (E. Javelle, S. Briolant);; French Armed Forces Center for Epidemiology and Public Health, Marseille (F. de Laval, A. Dia, C. Brossier, S. Sicard, V. Pommier de Santi);; Bégin Military Teaching Hospital, Saint Mandé, France (C. Ficko, A. Bousquet, A. Mérens, A. Courtiol);; Armed Forces Biomedical Research Institute, Brétigny-Sur-Orge, France (D. Delaune);; French Military Health Service, Djibouti City, Djibouti (H. Pommier, F. Gala, Q. Savreux, J.-P. Sanchez, F. Robin)

**Keywords:** Chikungunya virus, dengue virus, chikungunya, dengue fever, malaria, outbreak, Djibouti, Africa, vector-borne infections, arboviruses, viruses, parasites, military personnel

## Abstract

During 2019–2020, a chikungunya outbreak occurred in Djibouti City, Djibouti, while dengue virus and malaria parasites were cocirculating. We used blotting paper to detect arbovirus emergence and confirm that it is a robust method for detecting and monitoring arbovirus outbreaks remotely.

Djibouti is a semi-arid country bordered by Eritrea, Somalia, and Ethiopia. In the region, the main vector of chikungunya virus (CHIKV) and dengue virus (DENV) is the *Aedes aegypti* mosquito. The French Armed Forces are stationed in Djibouti City, where 70% of the country’s population live (total population ≈900,000). Military bases and housing are located in the urban area, and the entire French Defense Community (FDC), including service members, families, and civilian employees, comprise a population of 2,700. 

During July–October 2019, a large-scale chikungunya outbreak (41,162 suspected cases, 16 laboratory-confirmed cases, attack rate 12.3%) occurred in Dire Dawa, Ethiopia, 260 km from Djibouti City (Appendix Figure) (*1*). In a 2010–2011 survey in Djibouti City, although no epidemic has been reported since, 2.6% of the population had serologic evidence of CHIKV infection (*2*). Given the road, rail, and air connections between the 2 cities and the CHIKV-naive local populations, we estimated the likelihood of a CHIKV outbreak in Djibouti City to be highly probable. Patient management was challenging because dengue fever and malaria are endemic to Djibouti (*3*). 

We describe the comprehensive response implemented by the FDC to these multiple vectorborne diseases and evaluated the use of blood on blotting paper for arboviral diagnosis. With the consent of patients, we collected and anonymized epidemiologic and clinical data for diagnostic purposes. According to French regulations, because this outbreak was considered an immediate threat to public health, ethics approval was not required for this investigation. 

## The Study

**Figure 1:**
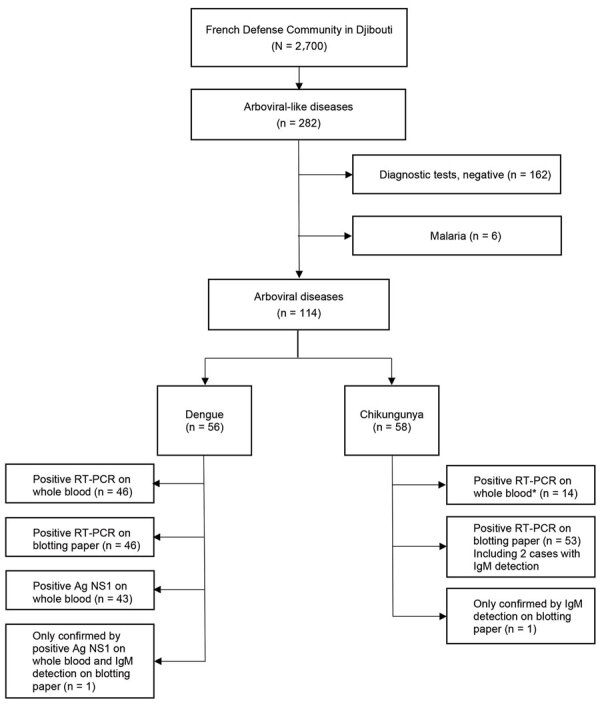
Flowchart for arboviral-like disease diagnoses among French Defense Community in Djibouti, 2019–2020. *Number of samples tested was limited because RT-PCR of whole blood was introduced in Djibouti City 1 month after the start of the chikungunya outbreak. Ag, antigen; NS1, nonstructural protein 1; RT-PCR, reverse transcription PCR**.**

In October 2019, we strengthened epidemiologic surveillance in the FDC to detect CHIKV emergence. We defined a suspected case of arboviral-like disease (ALD) as fever or chills and/or acute arthralgia and/or rash and/or vomiting and diarrhea. Symptomatic patients were encouraged to seek medical care for systematic testing for dengue, chikungunya, and malaria. From each person with ALD signs/symptoms, we collected venous blood, spotted it onto Whatman 3MM blotting paper (Sigma-Aldrich, https://www.sigmaaldrich.com), dried the samples at room temperature, and stored them in a sealed plastic pouch for preservation and transport (*4*). The National Reference Center for arboviruses in France performed reverse transcription PCR (RT-PCR) and serologic testing for CHIKV and DENV on blotting paper as described elsewhere (*5*). In January, equipment was set up locally to perform in-house RT-PCR for DENV and CHIKV on whole-blood samples (Figure 1) (*6*,*7*). Chikungunya cases were confirmed by positive RT-PCR on whole blood or blotting paper or by detection of CHIKV IgM on blotting paper. Dengue cases were confirmed by a positive DENV RT-PCR on whole blood or blotting paper or a positive nonstructural protein 1 (NS1) antigen rapid diagnostic test (RDT) (Bioline Dengue Duo; Abbott, https://www.abbott.com). We provided care according to the French National Recommendations (*8*) and World Health Organization guidelines (*9*). Concurrently, we strengthened the following in the FDC vector-control measures and personal protection: larval source management, long clothing, insect repellents, and long-lasting insecticidal nets. 

**Figure 2:**
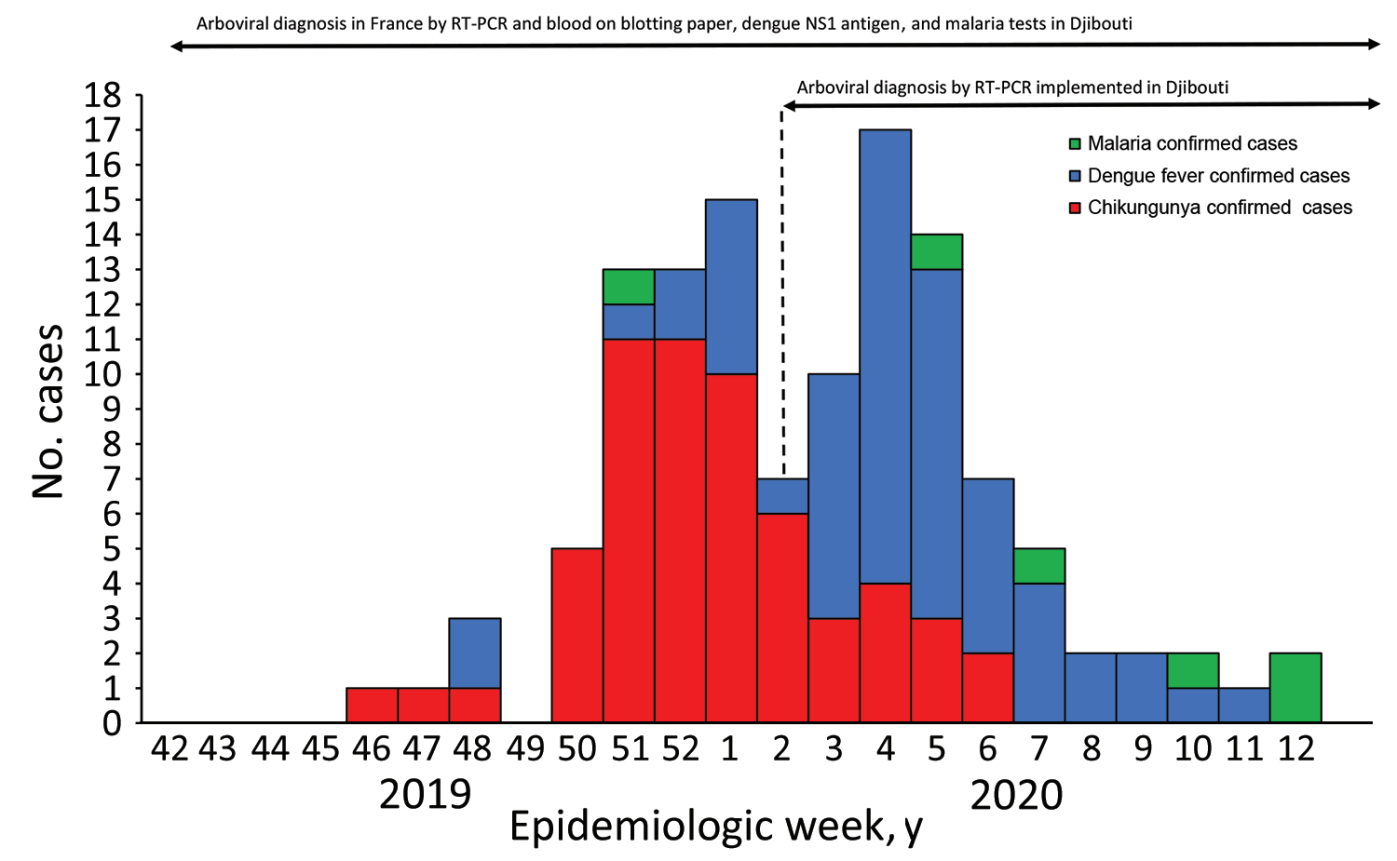
Vectorborne diseases among the French Defense Community in Djibouti: epidemic curve and availability of diagnostic tools, 2020 (chikungunya = 58, dengue = 56, and malaria = 6 cases). NS1, nonstructural protein 1; RT-PCR, reverse transcription PCR**.**

We compared clinical presentations of dengue and chikungunya by using R version 3.5.1 software (The R Project for Statistical Computing, https://www.r-project.org) for statistical analyses. Overall, among the 2,700 persons in the FDC, we included 282 with ALD. Through March 2020, we confirmed 120 cases of vectorborne disease (attack rate 42.6%, 120/282): 58 chikungunya (2.1%, 58/2,700), 56 dengue (2.1%), 6 malaria (5 *Plasmodium falciparum* and 1 *P. vivax*), and no co-infections (Figure 2). We also documented 2 concomitant influenza A virus and arbovirus infections. Among patients with vectorborne diseases, 67.5% (81/120) were male, and 73.3% (88/120) were service members. The median age was 34.5 (range 8.3–79.6, interquartile range 27.1–40.0) years, and 92.5% (111/120) of persons sought care within 48 hours of symptom onset (median 1, range 0–7, interquartile range 0–1 days).

We confirmed the first chikungunya case among persons in the FDC in November 2019. The outbreak started in December and lasted 13 weeks. The CHIKV strain belonged to the Indian lineage of the East/Central/South African genotype (*10*). The dengue outbreak peaked in late January and was linked to DENV-1 with a unique serotype, confirmed for 36/56 dengue cases (Figure 2). CHIKV and DENV co-circulated for 16 weeks. One chikungunya case was diagnosed by CHIKV IgM on blotting paper alone; all others (57/58, 98%) were confirmed by RT-PCR. One dengue case was diagnosed by positive NS1 antigen RDT with positive DENV IgM on blotting paper; all others (55/56, 98%) were confirmed by RT-PCR (Figure 1). The National Reference Center received blotting paper samples for 93.0% (106/114) of the DENV and CHIKV infections and confirmed 97.2% (103/106) of the diagnoses, 93.4% (99/106) by RT-PCR and 3.8% (4/106) by serology (1 DENV and 3 CHIKV). DENV and CHIKV RT-PCR testing were performed both on whole blood and on blotting paper for 44.7% (51/114) (Tables 1, 2). Compared with RT-PCR of whole blood, no RT-PCR of blotting paper produced false-positive results.

**Table 1:** Chikungunya cases confirmed by RT-PCR (n = 57/58) of blood samples, WB or BP, among 114 confirmed cases of arboviral disease, Djibouti*

Results	Positive on BP, no. (%)	Negative on BP, no. (%)	NA for BP, no. (%)	Total
Positive on WB	10 (72)	2 (14)	2 (14)	14
Negative on WB	0	39 (85)	7 (15)	46
NA for WB	43 (80)	11 (20)	0	54
Total	53 (46)	52 (46)	9 (8)	114

*RT-PCR tests were performed from October 2019 on dried blood spots stored on BP at the French National Reference Center for arboviruses in France. From January 2020, RT-PCR tests were also locally performed on WB samples at the French military medical center in Djibouti. BP, blotting paper; NA, not available (samples or result missing); RT-PCR, reverse transcription PCR; WB, whole blood.

**Table 2:** Dengue cases confirmed by RT-PCR (n = 55/56) of blood samples, WB or BP, among 114 confirmed cases of arboviral disease, Djibouti*

Results	Positive on BP, no. (%)	Negative on BP, no. (%)	NA for BP, no. (%)	Total
Positive on WB	37 (81)	2 (4)	7 (15)	46
Negative on WB	0	12 (86)	2 (14)	14
NA for WB	9 (17)	45 (83)	0	54
Total	46 (40)	59 (52)	9 (8)	114

*RT-PCR tests were performed from October 2019 on dried blood spots stored on BP at the French National Reference Center for arboviruses in France. From January 2020, RT-PCR tests were also locally performed on WB samples at the French military medical center in Djibouti. BP, blotting paper; NA, not available (samples or result missing; RT-PCR, reverse transcription PCR; WB, whole blood.

Samples from ALD patients were locally tested with NS1 antigen RDT, and 43 (43/120, 36%) results were positive. Results were negative for 13/56 (23%) persons with dengue, all tested within a mean delay of 1.5 (range 0–3) days from symptom onset. Among the 46 with DENV infection confirmed by whole-blood RT-PCR, 36 (78%) had concomitant positive RDT results.

The main ALD sign was fever (90.8%, 109/120). Headaches and digestive disorders were more associated with dengue fever (odds ratio [OR] 7.2, 95% CI 2.3–22.8) than chikungunya (OR 5.9, 95% CI 1.8–19.6) (Appendix Table).­ Highly predictive of chikungunya were arthralgia of the toe (OR 29.97, 95% CI 3.19–195.61), ankle (OR 18.28, 95% CI 6.14-54.71), finger (OR 12.47, 95% CI 3.93–39.61), and wrist (OR 18.27, 95% CI 5.71–58.52). Secondary infection developed in 4 patients with chikungunya (1 case each of pneumonia, dysentery, herpetic recurrence, and gingivitis with oral candidiasis). Among dengue patients, 4 had hepatic cytolysis (maximum transaminases elevation 12 times the upper limit), and 3 had secondary infections including acute pneumonia, *Escherichia coli* pyelonephritis, and intestinal amoebiasis. No patient met criteria for having severe dengue. No ALD patient required intensive care. All malaria patients recovered after a 3-day course of artenimol/piperaquine and secondary treatment with primaquine treatment for the patient with *P. vivax* infection. Treatment of arboviral disease relied essentially on analgesics, antihistamines, and hydration. The prescription of nonsteroidal anti-inflammatory drugs, aspirin, or corticosteroids was formally contraindicated during the first days of any infection. For patients with confirmed chikungunya, we carefully assessed the benefit-risk balance of introducing nonsteroidal anti-inflammatory drugs.

## Conclusions

Despite recent improvement in diagnostic tools, chikungunya outbreaks in Africa are probably underreported (*11*). During 2019–2020, a large-scale chikungunya outbreak occurred in Djibouti City (*12*). However, because of lack of diagnostic tests and dedicated reporting, no data are available to estimate its extent. The chikungunya outbreak remained limited (attack rate 2.1%) in the FDC but was followed by a dengue outbreak. We found that clinical features are helpful but not sufficient to discriminate between chikungunya and dengue (*13*,*14*). Biological confirmation remains necessary for determining appropriate care. The use of blood samples on blotting paper has been described as a field method for detecting arboviruses (*4*,*5*), routinely used in the French Armed Forces when deployed in Africa (*15*). In this study, we used blood samples on blotting paper to detect emergence of CHIKV and monitor the course of the outbreaks. Blotting paper provided a robust method for blood sampling and transport to a reference laboratory, making it possible to confirm 90% of the arboviral diagnoses. We recommend blotting paper as a field tool to detect and monitor arboviral epidemics remotely.

Appendix Supplemental information for study of chikungunya outbreak in a country with multiple vectorborne diseases, Djibouti, 2019–2020.
