# Supplementary material for: Chikungunya Outbreak in Country with Multiple Vectorborne Diseases, Djibouti, 2019–2020
Source: Emerg Infect Dis. 2023 Apr;29(4):826–30. doi: 10.3201/eid2904.221850 (PMC10045711; doi:10.3201/eid2904.221850)
Supplement: Appendix — Supplemental information for study of chikungunya outbreak in a country with multiple vectorborne diseases, Djibouti, 2019–2020. [file 22-1850-Techapp-s1.pdf]

Article DOI: https://doi.org/10.3201/eid2904.221850

EID cannot ensure accessibility for supplementary materials supplied by authors. Readers who have difficulty accessing supplementary content should contact the authors for assistance.

Chikungunya Outbreak in Country with Multiple Vectorborne Diseases, Djibouti, 2019–2020

Appendix

Appendix Table. Early clinical characteristics of CHIKV and DENV cases, bivariate analysis, N=114

Characteristic	CHIKV (N= 58), no. (%)	DENV (N=56), no. (%)	Total (N=114), no. (%)	p-value	OR [95% CI]
Fever	N=52	N=46	N=98		
Yes	48 (92)	43 (93)	91 (93)	1.000	0.84 [0.18-3.95]
No	4 (8)	3 (7)	7 (7)		
Headaches	N=55	N=47	N=102		
Yes	33 (60)	43 (91)	76 (75)	<0.001	0.14 [0.04-0.44]
No	22 (40)	4 (9)	26 (25)		
Cervicalgia	N=54	N=47	N=101		
Yes	23 (43)	9 (19)	69 (68)	0.012	3.13 [1.27-7.74]
No	31 (57)	38 (81)	32 (32)		
Dorsalgia	N=54	N=47	N=101		
Yes	9 (17)	3 (6)	12 (12)	0.136	2.93 [0.74-11.56]
No	45 (83)	44 (94)	89 (88)		
Lombalgia	N=54	N=47	N=101		
Yes	21 (39)	10 (21)	31 (31)	0.06	2.4 [0.97-5.72]
No	33 (61)	37 (79)	70 (69)		
Shoulder pain	N=54	N=47	N=101		
Yes	14 (26)	3 (6)	17 (17)	0.015	5.13 [1.37-19.19]
No	40 (74)	44 (94)	84 (83)		
Elbow pain	N=54	N=47	N=101		
Yes	11 (20)	3 (6)	14 (14)	0.049	3.75 [0.98-14.39]
No	43 (80)	44 (94)	87 (86)		
Wrist pain	N=54	N=47	N=101		
Yes	34 (63)	4 (8.5)	38 (38)	<0.001	18.27 [5.71-58.52]
No	20 (37)	43 (91.5)	63 (62)		
Finger pain	N=54	N=47	N=101		
Yes	29 (54)	4 (8.5)	33 (33)	<0.001	12.47 [3.93-39.61]
No	25 (46)	43 (91.5)	68 (67)		
Knee pain	N=54	N=47	N=101		
Yes	30 (56)	10 (21)	40 (40)	<0.001	4.62 [1.92-11.16]
No	24 (44)	37 (79)	61 (60)		
Ankle pain	N=54	N=47	N=101		
Yes	37 (69)	5 (11)	42 (42)	<0.001	18.28 [6.14-54.71]
No	17 (31)	42 (89)	59 (58)		
Toe pain	N=54	N=47	N=101		
Yes	19 (35)	1 (2)	20 (20)	<0.001	29.97 [3.19-195.61]
No	35 (65)	46 (98)	81 (80)		
Arthralgia	N=54	N=47	N=101		
Yes	46 (85)	18 (38)	64 (63)	<0.001	9.26 [3.57-24.04]
No	8 (15)	29 (62)	37 (37)		
Arthritis	N=54	N=47	N=101		
Yes	11 (20)	1 (2)	12 (12)	0.004	11.77 [1.46-95.03]
No	43 (80)	46 (98)	89 (88)		
Tenosynovitis	N=54	N=47	N=101		
Yes	7 (13)	0 (-)	7 (7)	0.014	-

Characteristic	CHIKV (N= 58), no. (%)	DENV (N=56), no. (%)	Total (N=114), no. (%)	p-value	OR [95% CI]
No	74 (87)	47 (100)	94 (93)		
Myalgia	N=55	N=47	N=102		
Yes	29 (53)	18 (38)	47 (46)	0.17	1.80 [0.81-3.96]
No	26 (47)	29 (62)	55 (54)		
Stiffness	N=54	N=47	N=101		
Yes	18 (33)	4 (8.5)	22 (22)	0.003	5.38 [1.67-17.33]
No	36 (67)	43 (91.5)	79 (78)		
Rash	N=58	N=50	N=104		
Yes	37 (64)	17 (34)	54 (50)	0.004	3.42 [1.55-7.56]
No	21 (36)	33 (66)	54 (50)		
Pruritus	N=55	N=47	N=102		
Yes	15 (27)	3 (6)	18 (18)	0.008	5.5 [1.48-20.41]
No	40 (73)	44 (94)	84 (82)		
Dysgeusia	N=55	N=47	N=102		
Yes	12 (22)	4 (8.5)	16 (16)	0.100	3.0 [0.90-10.04]
No	43 (78)	43 (91.5)	86 (84)		
Conjunctivitis	N=55	N=47	N=102		
Yes	14 (25.5)	11 (23)	25 (24.5)	1.00	1.12 [0.45-2.77]
No	41 (74.5)	36 (77)	77 (75.5)		
Swollen lymph nodes	N=55	N=47	N=102		
Yes	14 (25)	2 (4)	16 (16)	0.005	7.68 [1.65-35.87]
No	41 (75)	45 (96)	86 (84)		
Edema	N=55	N=47	N=102		
Yes	8 (15)	1 (2)	9 (8)	0.036	7.83 [0.94-65.12]
No	47 (85)	46 (98)	93 (92)		
Minor bleeding	N=55	N=47	N=102		
Yes	1 (2)	1 (2)	2 (2)	1.000	0.85 [0.05-14.00]
No	54 (98)	46 (98)	100 (98)		
Digestive disorders	N=55	N=47	N=102		
Yes	4 (7)	15 (32)	19 (18)	0.002	0.17 [0.05-0.55]
No	51 (93)	32 (68)	83 (82)		
Paresthesia	N=55	N=47	N=102		
Yes	6 (11)	0 (-)	6 (6)	0.029	-
No	49 (89)	47 (100)	96 (94)		

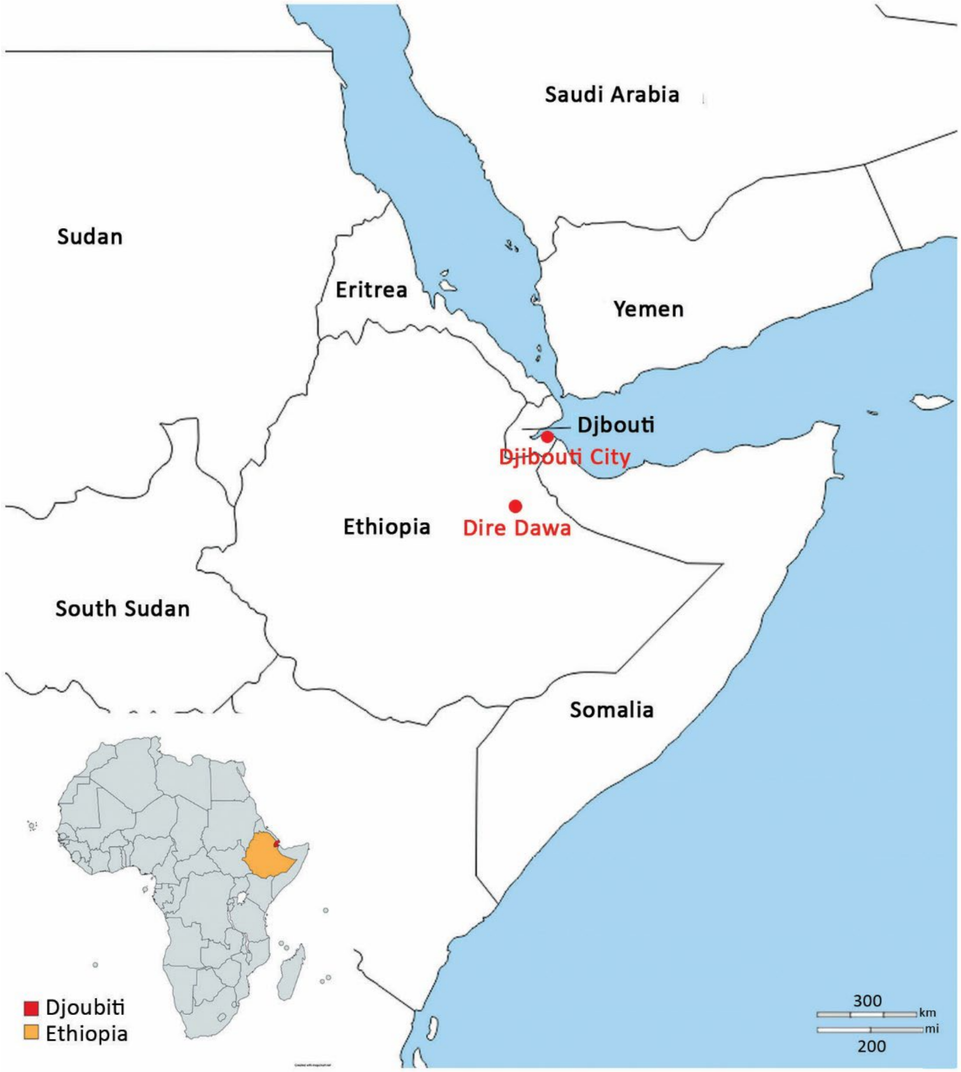

Appendix Figure. Location of Djibouti in the Horn of Africa. Chikungunya outbreaks hit the city of Dire Dawa in Ethiopia in October and Djibouti City in December 2019. The base map is available at https://d-maps.com/carte.php?num_car = 5504&lang = fr
